# Delocalization of Electrons in Strong Insulators at High Dynamic Pressures

**DOI:** 10.3390/ma4061168

**Published:** 2011-06-21

**Authors:** William J. Nellis

**Affiliations:** Department of Physics, Harvard University, Cambridge, MA 02138, USA; E-Mail: nellis@physics.harvard.edu; Tel.: +1-510-847-6821; Fax: +1-617-496-5144

**Keywords:** dynamic pressure, metallic liquid H, oxide metallic glasses

## Abstract

Systematics of material responses to shock flows at high dynamic pressures are discussed. Dissipation in shock flows drives structural and electronic transitions or crossovers, such as used to synthesize metallic liquid hydrogen and most probably Al_2_O_3_ metallic glass. The term “metal” here means electrical conduction in a degenerate system, which occurs by band overlap in degenerate condensed matter, rather than by thermal ionization in a non-degenerate plasma. Since H_2_ and probably disordered Al_2_O_3_ become poor metals with minimum metallic conductivity (MMC) virtually all insulators with intermediate strengths do so as well under dynamic compression. That is, the magnitude of strength determines the split between thermal energy and disorder, which determines material response. These crossovers occur via a transition from insulators with electrons localized in chemical bonds to poor metals with electron energy bands. For example, radial extents of outermost electrons of Al and O atoms are 7 a_0_ and 4 a_0_, respectively, much greater than 1.7 a_0_ needed for onset of hybridization at 300 GPa. All such insulators are Mott insulators, provided the term “correlated electrons” includes chemical bonds.

## 1. Introduction

Electrons in strong insulators are delocalized at high pressures. A strong insulator is defined to be one that has a high bulk modulus and a wide band-gap at ambient. A weak insulator is defined here to be one that has a small bulk modulus and a wide band-gap at ambient. Examples are sapphire (single-crystal Al_2_O_3_) and liquid H_2_, respectively. Their band-gaps at ambient are 10 eV and 15 eV, respectively. Sapphire is one of the most incompressible materials known and condensed H_2_ is one of the most compressible materials. Despite the great disparity in strength and compressibility, both of these wide band-gap insulators undergo a continuous transition or crossover to poor metals at ~100 GPa shock pressures and probably by the same general mechanism. The purpose of this paper is to discuss the likely reason why such different insulators would behave so similarly with respect to the onset of electrical conduction with essentially the same value of conductivity—minimum metallic conductivity.

### 1.1. Basic Idea under Dynamic Pressure

An electronic transition to a metallic state is called an insulator-metal transition (IMT). Many IMTs are associated with structural phase transitions or crossovers, as likely occurs in sapphire to an amorphous state at a shock pressure of ~300 GPa (3 million bar = 3 Mbar) [1] and does occur in liquid H_2_ at 140 GPa [2]. While one would normally not expect materials with such an extreme difference in strength to have much in common in terms of an IMT, the metallization processes in these two at high shock pressures are probably extreme limits of the same general process.

One key to recognizing this situation is the fact that crossovers to poor metals in both fluid hydrogen and amorphous Al_2_O_3_ are accomplished by dynamic or shock compression. Shock compression generally means compression by a single shock wave; dynamic compression is a more general term that often means compression by multiple shock waves or by ramp waves in time. In this materials context, the difference is a detail. The important point with respect to materials is that shock and dynamic compression are dissipative and dissipation in shock flows drives atomic disorder and electron delocalization in amorphous solids and in fluids.

Shock compression is dissipative, in the sense that not all the energy deposited by shock compression goes into compression; *i.e.*, into PdV work, where P is pressure and V is volume. Dissipation energy goes into temperature T, or non-equilibrated thermal energy, and entropy S, thermally equilibrated or not. Entropy is a word used here to denote the concept of damage or disorder. Shock-induced IMTs have been observed at elevated shock temperatures (T ≥ 1,000 K) and are thus often called semiconductor-metal transitions (SCMT).

The basic idea is that at sufficiently high dynamic pressure there is sufficient dissipation in the form of thermal energy and entropy to destroy chemical bonds, which localize electrons at ambient. Once chemical bonds are destroyed with high dynamic pressures, wave functions of disordered atoms overlap at sufficiently high densities for itinerant energy bands to form. In Al_2_O_3_ and H_2_ the bonds are Al-O and H-H and both bonds have the same bond strength, 4.5 eV [3]. On the other hand, their material structures are totally different. At low pressures Al_2_O_3_ has a rigid, three-dimensional (3D), ordered crystal structure with a strong bond between all Al-O pairs in the 3D lattice. In contrast liquid H_2_ is a disordered structure in which H_2_ molecules interact via a weak Vander Waals pair potential with a well-depth of 0.004 eV [4].

The different initial structures significantly affect the way these two materials respond on a microstructural scale to dynamic pressure. However, in both cases chemical bonds eventually dissociate under sufficiently high dynamic pressure. Once the bonds are destroyed, electrons that were correlated in local chemical bonds have spherically symmetric or spherically-averaged atomic wave functions that hybridize into itinerant energy bands of a metal at sufficiently high densities. Shock pressures in the 100 GPa range produce sufficiently high densities. This process is termed Bonds to Bands (BtB). Material strength determines the split between thermal energy and entropy, but not whether or not a BtB can occur. Because of the highly disordered metallic state, conduction electron scattering is strong and so “metallic conductivity” means minimum metallic conductivity (MMC ≈ 2,000/(Ω-cm) = 500 µΩ-cm.), as observed in fluid H_2_ [2] and as predicted based on modest extrapolation of measured electrical conductivities of Al_2_O_3_ shock-compressed up 220 GPa [1].

### 1.2. Possibilities under Static Pressure

Several strong insulators also probably reach MMC in a diamond-anvil cell (DAC) at 100 GPa static pressures at 300 K. These are ones with frustrated phase transitions that produce amorphous samples at sufficiently high static pressures. An example is Al_2_O_3_, which has been compressed in a DAC up to 200 GPa [5]. In the as-compressed state, only a disordered α-corundum phase was observed up to 180 GPa in a DAC. Laser heating up to ~2,000 K was required at high pressures in order to drive and then thermally quench high-pressure phases to ambient temperature at high pressure. For the Al_2_O_3_ sample compressed to 200 GPa, no crystalline symmetry was reported, which presumably means the Al_2_O_3_ sample compressed statically to 200 GPa was amorphous. It is possible that amorphous Al_2_O_3_ reaches MMC at ~300 GPa in a DAC, as suggested previously [1]. A similar situation occurs in Gd_3_Ga_5_O_12_ (GGG) [6,7,8], as discussed below.

## 2. Mott-Like Insulators

The notion that chemical bonds in electrical insulators localize electrons and that electron delocalization is achieved by breaking those bonds to form metallic energy bands is similar to the idea of a Mott insulator, of which NiO is a paradigm [9]. Based on its band structure, NiO is expected to be a metal. However, it is actually a transparent, anti-ferromagnetic insulator. Mott proposed that many-body electron correlations localize electrons at ambient, and that an IMT probably occurs at high pressures. A chemical bond consists of correlated-electron wave functions and their associated correlated electron spins. The cases considered here involve nonmagnetic insulators, which traditionally have not been considered to be Mott insulators. To distinguish classical magnetic Mott insulators from ones with shock-induced SCMTs in nonmagnetic crystals, I call the latter ones Mott-like. However, Mott and Mott-like insulators are essentially the same, provided the definition of Mott insulator is generalized to include all insulators with electron correlations that localize electrons at ambient, magnetic or not, and become metallic at high pressures.

## 3. Dynamic Compression

Extreme states of condensed matter are generated via the coupling between shock hydrodynamics and either equilibrium thermodynamics or non-equilibrium microstructures. Pressures used in experiments discussed herein were single-shock pressures in the case of Al_2_O_3_ and multiple-shock compression in the case of H_2_. Shock pressures were generated by high-velocity (1 to 8 km/s) impacts of a planar solid disc onto a sample target at rest. Experimental lifetimes were typically ~100 ns. Rise times of shock pressures ranged from a few tenths to a few tens of ns. Because of the fast time scale, the method is called dynamic compression. Because of the 100 ns lifetime, dynamic compression is adiabatic (too brief for heat to diffuse out of a sample), too brief for highly-mobile shock-compressed and heated hydrogen to diffuse out of its sample holder, sufficiently long for fluids and metals to equilibrate thermally, and too brief for strongly covalently-bonded materials to equilibrate thermally. A general discussion of dynamic compression has been published [10].

In this paper we present arguments for the fact that metallization of all insulators at high dynamic pressures probably occurs via a single general mechanism. Strong and weak insulators, such as sapphire and liquid H_2_, are two extreme cases of this general mechanism and these extreme cases facilitated its recognition. In Section 4 the classical IMT in H_2_ is discussed. In Section 5 the SCMT in fluid hydrogen is discussed because it is the first experimental observation of a metallic phase of highly condensed hydrogen, is a reference point for discussion of metallization of strong insulators, and as an illustration of how shock dissipation causes the Bonds-to-Bands Transition (BtBT) in H_2_. It is shock dissipation that enabled metallic fluid H to exist at pressures sufficiently low to be observed in a laboratory. The IMT from solid H_2_ to metallic hydrogen at low temperatures (T < 300 K) is yet to be observed experimentally. In Section 6 general shock synthesis of amorphous metallic oxides is discussed. Experimental results for Al_2_O_3_ and Gd_3_Ga_5_O_12_, are reviewed, which includes comparing radial extents of electron charge-densities of the various atoms to the size of the average volumes into which they must fit at high pressures. The latter provides a basis for determining if a material is likely to be metallic at a given compression. Basic conclusions are given in Section 7.

## 4. Classical IMT in H_2_

The IMT from H_2_ to H at T = 0 K was proposed by Wigner and Huntington (WH) in 1935 [11] and remains today the paradigm of a pressure-driven first-order phase transition. In particular, WH demonstrates the intrinsic importance and simplicity of the dissociative phase transition for achieving a metallic state. The classical IMT of WH was a paradigm for understanding the crossover under dynamic compression from insulating liquid H_2_ to metallic fluid H [2], which in turn provided a basis for understanding the likely crossover from strong insulators to disordered poor metals [1]. So as background for discussions below, we begin with a discussion of the WH IMT in H_2_.

WH’s classical view of an IMT under pressure is one of a crystalline insulator compressed hydrostatically at temperature T = 0 K and at some high pressure a first-order dissociative phase transition to a metallic state occurs [11]. H_2_ is an insulator because two electrons are localized in each intramolecular H-H bond. However, on dissociation to H one electron per atom at sufficiently high density means one electron per energy band, which is a band structure of a metal. The dissociative phase transition from H_2_ to metallic H occurs simply because of dissociation and the resulting half-filled energy band, independent of band shape.

In 1935 WH predicted theoretically the classical IMT in H_2_ would occur at an estimated pressure of ~25 GPa. The IMT in solid H_2_ has yet to be observed experimentally under static pressures up to ~300 GPa. Optical studies on solid H_2_ in a DAC suggest metallic H_2_ should be observed at about 450 GPa near 100 K [12]. The current theoretical estimate of pressure for the dissociative transition from solid H_2_ to solid metallic H at T = 0 K is 500 GPa [13]. Both of these predicted pressures are well beyond current capabilities of a diamond anvil cell (DAC).

It is theoretically possible, in principal, for H_2_ to metalize at T = 0 K if its energy gap closes to k_B_T = 0, where k_B_ is Boltzmann’s constant. However, H_2_ dissociation energy and band gap near ambient are 4.5 eV [3] and 15 eV [14], respectively. While the density-dependences of these parameters are not yet known exactly, the fact that the band gap at ambient is a factor of 3.3 times larger than dissociation energy, implies that dissociation at T = 0 K, and thus metallization, is more likely to occur at a lower density/pressure than band-gap closure of H_2_. Thus, the IMT in H_2_ probably occurs by dissociation, though this point is yet to be answered by experiment. One likely reason the IMT of WH is yet to be observed is the large H-H bond strength of 4.5 eV, which is also the strength of the Al-O bond. To date it has not been possible to deposit 4.5 eV into solid H_2_ to dissociate it by compression alone at T = 0 K.

## 5. Shock Dissipation in Liquid H_2_: Metallic Fluid H

Since predicted pressures required to metalize hydrogen in a DAC at low temperatures are beyond current DAC capability, it was appropriate to look for the IMT in H_2_ by trying something in addition to pure compression, something that might induce the IMT in H_2_ at pressures that can be achieved in a laboratory. Heating compressed H_2_ in a DAC is the obvious choice because heating might drive dissociation, which produces metal. However, if H_2_ at ~100 GPa in a DAC is heated above ~300 K, H_2_ diffuses out of a DAC in a few minutes, which thus far has been a major impediment to making metallic hydrogen in a DAC.

So the issue then becomes the more complex one of finding a method at finite T. The free energy F of a system is F = U−TS, where U is internal energy and dissipation energy is E_d_ = TS. The method used would need to compress condensed hydrogen adiabatically to a temperature T with a ~100 GPa pressure pulse to increase its density by a factor of ~10 in an experimental lifetime such that hydrogen has insufficient time to diffuse out of the sample holder but sufficient time to thermally equilibrate to T and S. In H_2_, dissociation is the dominant contribution to ΔS. Because the method must be adiabatic, pressurization, compression, and heating must be simultaneous. Irreversible shock energy deposited in the multiple-shock compression process is divided between T and S such that their product is maximized in order to minimize the free energy. Thus, at sufficiently large T > 0 and entropy increase ΔS > 0, the SCMT in H_2_ might be achieved to a metallic H phase at lower pressures than can be achieved at T = 0 K at the same density.

Dynamic compression achieves ~100 GPa pressures, up to 10-fold compression of initial density of liquid H_2_, at temperatures up to ~3,000 K simultaneously for experimental lifetimes of ~100 ns. Because of the fast rise time of pressure (~10 ns) and the short experimental lifetime, dynamic compression is dissipative and adiabatic, respectively. That is, experimental lifetime is too short for heat and hydrogen to diffuse out of the compressed hydrogen sample. Shock dissipation energy E_d_ goes into T and S. This SCMT has in fact been observed experimentally. By simultaneously pressurizing, compressing, and heating liquid H_2_ initially at 20 K with multiple-shock compression, MMC of liquid H is achieved at 140 GPa, 9-fold compressed initial liquid-H_2_ density, and ~3,000 K [2,15].

Ross has shown this SCMT is facilitated by the fact that H_2_ dissociation energy decreases with compression [16]. At the same time temperature increases with dynamic compression. Thus, at a sufficiently high dynamic pressure, H_2_ molecules dissociate to metallic fluid H in a crossover region. The H temperature of 3,000 K is much higher than melting temperatures of H_2_, measured experimentally and calculated theoretically at 140 GPa [17,18,19,20,21]. Thus, dense metallic H is a fluid. Because of the large compression, the Fermi temperature T_F_ exceeds 200,000 K, T/T_F_ ~ 0.01, and metallic fluid H is highly degenerate. The highest measured value of electrical conductivity in the fluid is 2,000/(Ω-cm) at pressures from 140 to 180 GPa, which is MMC and consistent with theory of dense hydrogen [22,23].

Dissociation of the H-H bond achieves spherically symmetric H atoms. Sufficient pressure was available in those experiments to achieve sufficient overlap of electronic wave functions on adjacent H atoms to form an itinerant energy band of a metal. N_2_ and O_2_ undergo a similar crossover from a diatomic insulator to monatomic metallic fluid. Fluid H, N, and O have similar measured values of MMC (2,000 (Ω-cm)^−1^) at highest pressures (100 to 140 GPa) [2,24,25,26]. Because T is finite, at dynamic pressures lower than required to achieve MMC, hydrogen, nitrogen, and oxygen are semiconductors. Expanded-fluid Cs and Rb near their liquid-vapor coexistence curves at ~2,000 K reach MMC at ~10 MPa (100 bar) static pressures [27]. Thus, five elemental monatomic fluids, H, N, O, Rb, and Cs, undergo a Mott-like SCMT under pressure.

**Figure 1:**
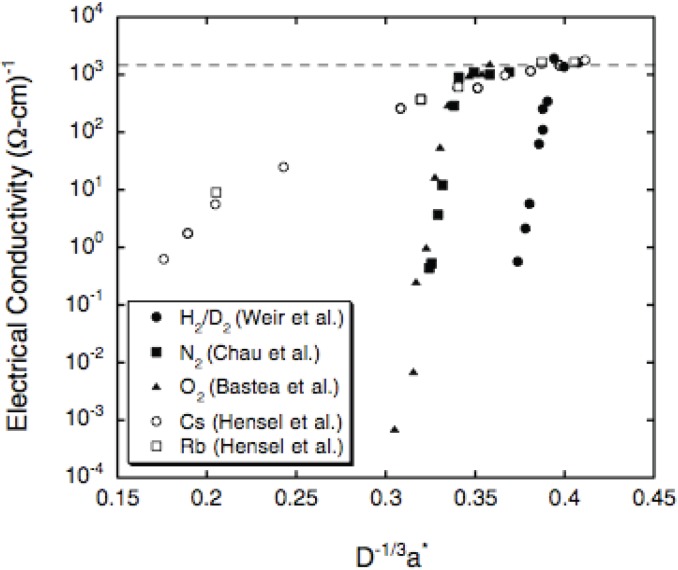
Electrical conductivities of H, N, O, Rb, and Cs plotted *versus* a*/D^1/3^, where a* is size of atom (effective Bohr radius) and D is volume of average cube around each atom. D^1/3^ is average distance between adjacent nuclei. Each point is measured electrical conductivity at specific pressure [24].

Figure 1 is a plot of electrical conductivities of H, N, O, Rb, and Cs *versus* a*/D^1/3^, the ratio of atom size, assumed to be the effective Bohr radius a*, to the average distance between adjacent atoms in the fluid, which is determined by density and thus by pressure. D is the volume of the average cube around each atom. D^1/3^ is average distance between adjacent nuclei. Plotting data this way for systems near an IMT was suggested by Mott [28], and applied by Hensel to his Rb and Cs conductivity data [27]. Figure 1 implies the radial extents of wave functions of Rb and Cs are relatively large because relatively little compression of Rb and Cs is required for them to conduct. Similarly, H requires substantial compression in order to conduct. N and O are intermediate but closer to H. Figure 1 also shows that as atoms are pushed together by pressure, conductivity increases until at highest pressures all five elements have MMC (2,000 (Ω-cm)^−1^). Since a*/D^1/3^~0.35–0.38 for 5 elements at MMC, overlap of wave functions on adjacent atoms is substantial for all of them. The maximum value a*/D^1/3^ can have is 0.5, which corresponds to coincidence of maxima in electron densities on adjacent atoms. The idea that large overlap is also probably required for oxides to become poor metals at high pressures arises below in the discussion of Al_2_O_3_ and Gd_3_Ga_5_O_12_.

The trend above is as expected. For example, attraction by the Cs nucleus of the outer 6s^1^ conduction electron is screened by a Xe core. Substantial screening means the radial extent of the 6s^1^ electron is relatively large and so relatively little compression is needed to obtain sufficient overlap of wave functions on adjacent sites to cause the onset of electrical conduction. For H, the opposite is true. The attraction between electron and proton is unscreened and so substantial compression is expected before the onset of conduction in fluid H. Screening in N and O is relatively small compared to that in Cs. That is, screening in N and O is caused by filled 1s^2^ and 2s^2^ electron shells, which are relatively small compared to the size of a Xe core in Cs. Thus, onset density of conductivity in N and O is closer to that in H.

**Figure 2:**
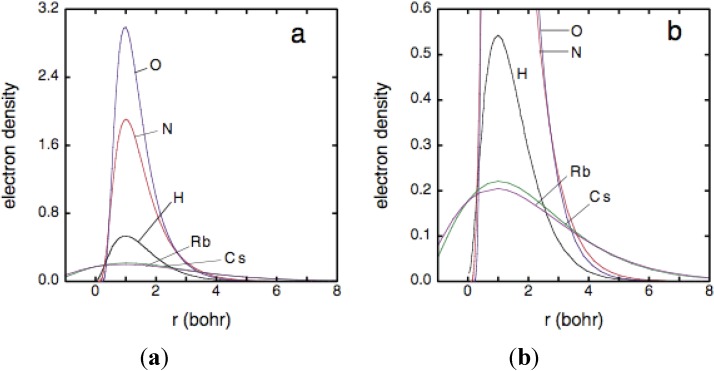
Electron densities (4πr^2^ψ*ψ) plotted *versus* radii [24,29]. To look at radial extents of outer electrons, for comparison purposes peak of each distribution was shifted to r = 1 bohr in plots. Curves in (**b**) are curves in (**a**) plotted on expanded scale.

Statements about expected radial extents of atomic wave functions derived from Figure 1 are readily checked by comparing radial electron-density distributions calculated in the Hartree-Fock-Slater approximation for the five elements [24,29]. These results are shown in Figure 2. Inspection of (b) shows that the radial extent of the electron density distribution for H is least, greatest for Rb and Cs, and intermediate for N and O as well as being closer to that of H than to that of Rb and Cs, as expected.

Fluid H, N, O, Rb, and Cs have been discussed in some detail to demonstrate that Mott plots of measured electrical conductivities *versus* Mott parameter a*/D^1/3^ provide qualitative estimates of the relative radial extents of electron density distributions, calculated in the Hartree-Fock-Slater approximation. This extensive self-consistency between experimental results and atomic structure calculations has two significant implications. First, it is reasonable to use radial electron-density distributions of atoms calculated in the Hartree-Fock-Slater approximation to make estimates about densities at which materials more complex than simple fluids might be metallic at extreme conditions. In this regard, likely metallization pressures and densities of Al_2_O_3_ and Gd_3_Ga_5_O_12_ are discussed in the next section. Second, electrical conductivities of elemental fluids measured with a 20-m long two-stage light-gas gun enable resolution of radial extents of quantum mechanical electron density distributions on the spatial scale of ~Bohr.

If metastable solid metallic hydrogen (MSMH) could be quenched to ambient pressure and temperature, this material might have several scientific and technological uses including: a quantum solid with novel physical properties, including room-temperature superconductivity; a very light-weight structural material; a chemical fuel, propellant, or explosive, depending on the rate of release of stored energy; a dense nuclear-fusion fuel made with isotopes deuterium and tritium, rather than hydrogen, to obtain higher energy yields in inertial confinement fusion [30].

In summary, under dynamic compression, “soft” fluids, such as H_2_ rapidly undergo large compressions, which induce high dynamic temperatures. In such systems thermal equilibrium is generally achieved in a sub-ns time scale. As dynamic pressure increases H_2_ dissociates which causes entropy to increase and thermally equilibrate also on a sub-ns time scale. Thus, dynamically compressed fluids can be treated with equilibrium thermodynamics. In this case relatively simple assumptions and approximations give theoretical results in generally good agreement with experiment. Representative examples are Ross’ work on dissociation of H_2_ [16] and Mott’s MMC [28].

## 6. Shock Dissipation in Strong Insulators: Likely Synthesis of Metallic Oxide Glasses

Strong insulators differ substantially in most respects from weak fluid insulators discussed in Section 5. Interaction potentials differ substantially, breaking strong bonds in a rigid 3D lattice requires several eV, and atom densities in strong insulators near ambient are quite high compared to fluids. As a result, up to 10–100 GPa shock pressures, shock dissipation in dense strong insulators is absorbed substantially by mechanically breaking and bending inter-atomic bonds. In fact in this range of shock pressures, shock-induced damage and heating are often heterogeneous and T and S do not equilibrate thermally in bulk during experimental lifetimes until shock pressures exceed ~100 GPa. Nevertheless, with modest extrapolations of existing conductivity data of Al_2_O_3_ and Gd_3_Ga_5_O_12_ up to ~250 GPa, both likely reach MMC at 300–400 GPa, as do compressible fluid H, N, and O at ~100 GPa.

### 6.1. Al_2_O_3_ (Sapphire)

Sapphire with a density of 3.98 g/cm^3^ disorders substantially under shock pressures up to ~100 GPa ([31] and references therein). It is likely that shock heating of Al_2_O_3_ is not uniform in bulk until shock pressures exceed ~200 GPa. Moreover, the Hugoniot and 300-K isotherm of sapphire are nearly coincident up to 400 GPa. Above ~400 GPa, Hugoniot pressure increases dramatically and diverges from the 300-K isotherm. These observations suggest a picture in which entropy dominates dissipation below ~400 GPa and once entropy is maximized total pressure and thus shock temperature and thermal pressure increase rapidly with additional shock compression.

In a DAC, the corundum (α-Al_2_O_3_) to Rh_2_O_3_(II)-type phase transition occurs at 103 GPa and the Rh_2_O_3_(II)-type to CaIrO_3_-type transition occurs at 130 GPa and persists to at least 180 GPa. However, these transitions in a DAC are sluggish. They occur only with laser-heating and thermal quenching at high pressures. X-ray spectra of laser-heated and quenched samples consist of broad individual diffraction peaks superimposed on a significant broad background, indicative of disordered structures with short-range order [5]. Those samples in a DAC are substantially disordered, which means a substantial amount of entropy.

**Figure 3:**
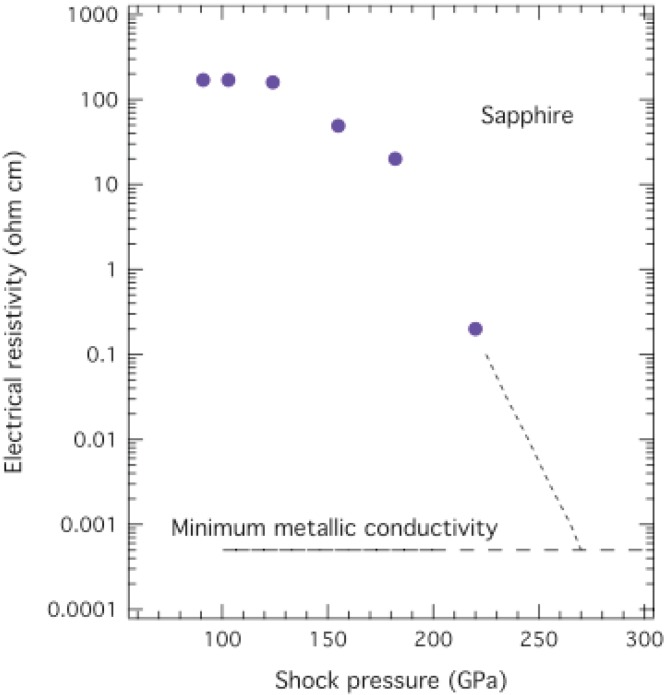
Measured electrical resistivity of sapphire plotted *versus* shock pressure up to 220 GPa [32].

The effect of shock-induced disorder on measured electrical resistivities of sapphire at shock pressure from 91–220 GPa [32] is illustrated in Figure 3. Up to ~130 GPa electrical resistivity is large and essentially constant. From 130–220 GPa, resistivity decreases by a factor of 10^3^ and extrapolates to 500 µΩ-cm around 280 GPa. 500 µΩ-cm corresponds to electrical conductivity of 2,000 (Ω-cm)^−1^, which is MMC, the same MMC as metallic fluid H reaches at 140 GPa, 9-fold compressed liquid-H_2_ density, and ~3,000 K (Section 5). In sapphire a static pressure of 130 GPa is the pressure of the Rh_2_O_3_(II)-type to CaIrO_3_-type transition in a DAC, and CaIrO_3_-type disorders increasingly at higher pressures in a DAC. The picture that emerges from all these experiments is that shocked sapphire damages in the corundum (α-Al_2_O_3_) and Rh_2_O_3_(II)-type phases; from 130–280 GPa shocked sapphire undergoes a crossover with increasing disordering. At ~280 GPa shocked sapphire has MMC, which suggests it is an amorphous atomic metal or metallic glass, basically a frozen fluid. Of course, the possibility exists that sapphire is a fluid at ~280 GPa because the melting curve of sapphire has yet to be measured at these pressures. Alternatively, Al and O might phase separate at these extreme conditions, which might mean that current is conducted through Al filaments surrounded by non-conductive oxygen. This is conceivable because Al composition is 40 at.% which exceeds the percolation limit for filamentary conduction.

A key issue to consider is whether or not it is possible for Al and O to form hybridized energy bands and, thus, have a band structure of a disordered Al-O metallic alloy. Chemical bonds have a characteristic length and energy. Shock compression is about a factor of 1.6 in density, which would shorten and distort bond lengths. Shock dissipation supplies energy to substantially disorder strong insulators. So it is reasonable to assume that all bonds are broken at sufficiently high shock pressures, which produces a disordered collection of Al and O atoms whose wave functions might hybridize. At shock compression of 1.6 in density and under the assumption that both Al and O atoms must fit into an average-size cube with the same volume, then each atom must fit into a cube with an edge length of 3.3 a_0_, where a_0_ is the Bohr radius. Thus, if the radial extents of the electron density distributions of Al and O atoms exceed 1.7 a_0_, then wave functions on adjacent atoms will overlap and it will be possible for them to hybridize in the band structure of a disordered solid solution.

**Figure 4:**
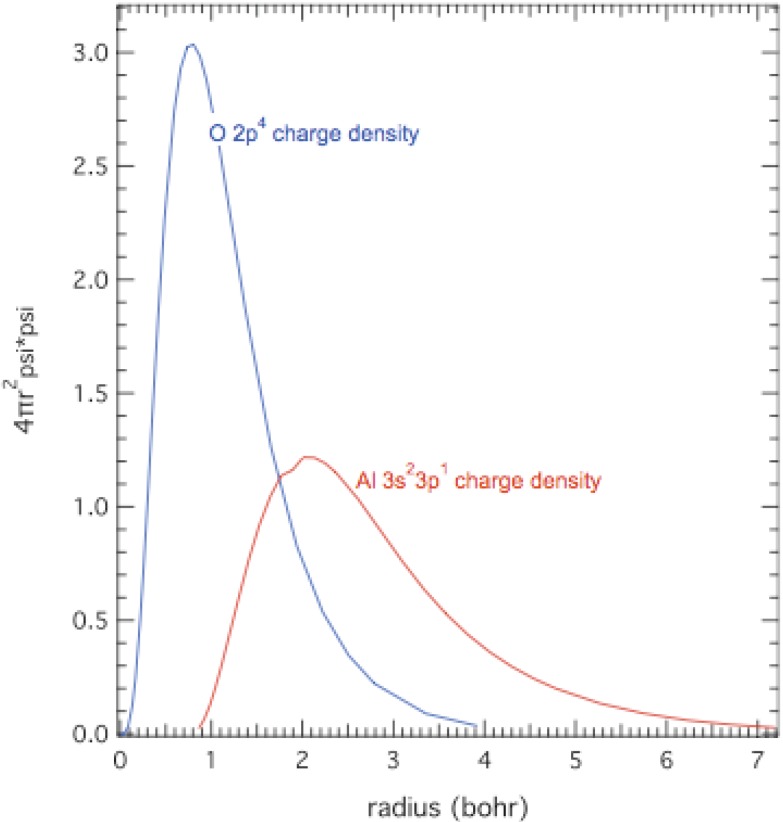
Spherically averaged atomic electron densities *versus* radius for O *2p*^4^ and Al *3s*^2^*3p*^1^ electrons calculated with Hartree-Fock-Slater method [1,29].

Radial extents of the outermost electrons of Al (3*s*^2^3*p*^1^) and O (2*p*^4^) atoms were calculated in the Hartree-Fock-Slater approximation [1,29] and are shown in Figure 4. The radial extents of outermost electrons of Al and O atoms are 7 a_0_ and 4 a_0_, respectively, both of which are much greater than 1.7 a_0_ needed for onset of hybridization. Overlap would be substantial, as in the case of compressible fluids discussed in Section 5. Thus, it is spatially possible for Al and O to form hybridized energy bands of a metal in such a dense atomic glass. Thus it is possible that MMC at ~280 GPa in Figure 3 is caused by hybridization of Al and O wave functions and strong electron scattering in an amorphous metallic alloy.

**Figure 5:**
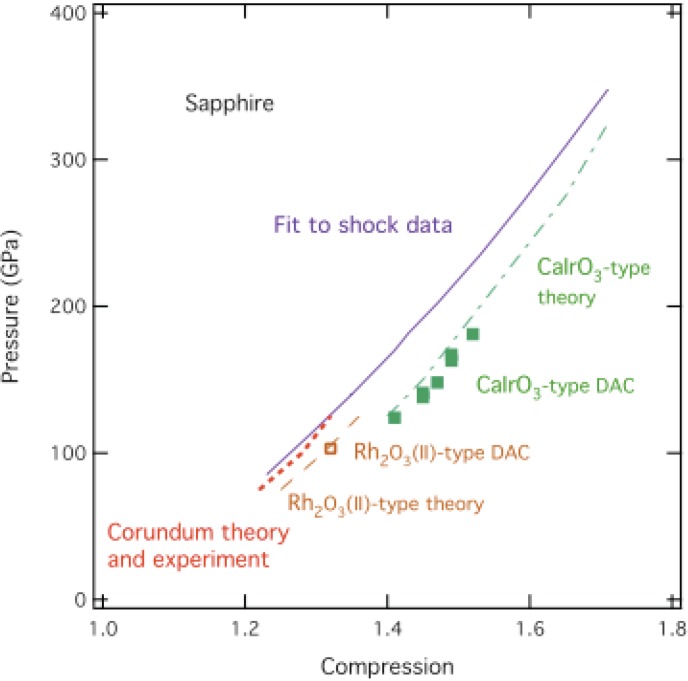
Pressure *versus* compression on shock Hugoniot [33], DAC data on 300-K isotherm [5], and theory [34] for Al_2_O_3_ up to 340 GPa.

Figure 5 compares the Hugoniot [33], DAC data on 300-K isotherm [5], and theory [34] for Al_2_O_3_ up to 340 GPa. Static compression to all data points shown produced only disordered corundum [5]. Laser-heating and thermal quenching at high pressures to 300 K were required to obtain those data points in Figure 5. Static compression of Al_2_O_3_ without a pressure medium and without laser-heating might produce a sample in a DAC with similar disorder as states on the Hugoniot at comparable compression. In this way it might be possible to synthesize an amorphous Al_2_O_3_ sample in a DAC for characterization. An amorphous Al_2_O_3_ sample in a DAC might have MMC at ~300 GPa, as well as under shock compression.

### 6.2. Gd_3_Ga_5_O_12_ (GGG)

GGG, with a density of 7.10 g/cm^3^ and no long-range magnetic order, disorders substantially under static and shock compression. In a DAC, GGG is crystalline up to 74 GPa, above which x-ray diffraction peaks broaden up to 84 GPa, above which GGG is amorphous [6]. Hugoniot data of GGG single crystals have been measured from 30 to 260 GPa. The Hugoniot elastic limit (HEL) of GGG was found to be 30 GPa, a transition to an intermediate (IM) phase occurs at 65 GPa and extends up to 120 GPa, followed by the onset of a virtually incompressible high-pressure (HP) phase that extends up to 260 GPa. Calculated shock temperatures of GGG reach 6500 K at 260 GPa. Up to ~70 GPa the pressure-volume data measured in a DAC at 300 K and the Hugoniot are virtually identical. From shock pressures of 120 up to 260 GPa (HP phase), electrical conductivity measurements show that GGG is semiconducting [7].

The sharp increase in slope of shock pressure *versus* compression of GGG at ~120 GPa and ~1,000 K [7] could be caused by a transition to a virtually incompressible phase (a density effect) or by onset of substantial shock temperature and thus thermal and total pressure. Mao *et al*. found that GGG becomes amorphous in a DAC at 88 GPa and transforms to a new high-pressure phase at 88 GPa on laser heating to 1500 K [8]. The high-pressure phase in a DAC is cubic, consistent with a perovskite structure and stoichiometry of (Gd_0.75_Ga_0.25_)GaO_3_, and persists up to 180 GPa at 1,500 K. No rapid increase in pressure with compression is observed up to 180 GPa in the DAC data. The DAC data imply the rapid increase in shock pressure with compression at 120 GPa is caused by a combination of fast incomplete phase transitions and increasing shock heating, which is consistent with observed semiconductivity. It is interesting to note that Al_2_O_3_, as well as GGG, must also be laser heated at 100 GPa pressures in a DAC to induce high-pressure crystalline phases [5].

Time-resolved shock-wave profiles and Hugoniot data of single-crystal GGG have also been measured from 8.5 to 113 GPa [35]. The HEL increases from 8 to 24 GPa as final shock pressure increases from 8.5 to 89 GPa. Such a strong pressure dependence of the HEL has not been reported previously and suggests metastability and disorder during experimental lifetimes at those pressures. The phase transition observed at 76 GPa is probably the one observed by Mashimo *et al.* at somewhat lower pressure. It is interesting to note that single-crystal Al_2_O_3_ also has very unusual shock-wave profiles as GGG at comparable shock pressures, which are also indicative of disorder [36].

Extrapolation of electrical conductivities of GGG measured up to 260 GPa suggest GGG reaches MMC at ~400 GPa (0.4 TPa). In order to determine if this metallization is reasonable, once again the radial extents of atomic charge densities were compared to the size of the average cube into which each must fit at pressure. The highest pressure phase of GGG in a DAC is cubic with an effective stoichiometry of (Gd_0.75_Ga_0.25_)GaO_3_. As for Al_2_O_3_, radial extents of atomic charge densities of outermost electrons of Gd (5d^1^6s^2^) and Ga (4s^2^4p^1^) were calculated with the Hartree-Fock-Slater method. Atomic Gd has a radial charge density distribution that is more extended with a lower broad maximum than that of Ga, which is very similar to that of Al. We assume that at 0.4 TPa and compression of 1.9 over initial crystal density, bonds in crystalline Gd_3_Ga_5_O_12_ are broken in a glass and Gd, Ga and O atoms can be treated as spherically averaged and symmetric. Calculated radial extents of 5d^1^6s^2^, 4s^1^4p^2^, and 2*p*^4^ electrons of Gd, Ga and O atoms are 8 a_0_, 7 a_0_, and 4 a_0_, respectively. These wave functions must fit into an average cube with an edge length of 3.5 a_0_, which means the distance from the center of the cube to a face is 1.8 a_0_, which is substantially less than the radial extents of outer electrons Gd, Ga, and O. Thus, substantial overlap of atomic wave functions occurs at 0.4 TPa in GGG and it too is likely a metal by band overlap at these extreme conditions.

## 7. Conclusions

Dissipation in shock flows at high dynamic pressures drives structural and electronic transitions or crossovers, such as to metallic liquid hydrogen and most probably Al_2_O_3_ and Gd_3_Ga_5_O_12_ metallic glasses.

These crossovers occur via Mott-like transitions from chemical bonds in which electrons are localized to disordered metallic systems in which electrons hybridize into itinerant energy bands and have MMC by virtue of strong electron scattering.

Dissipation is energy that does not go into compression at pressure-thermal energy and disorder or temperature T and entropy S if in thermal equilibrium. Contrary to popular opinion, dissipation is more than shock heating and significant shock heating is not required to make a poor metal.

The term “metal” here means electrical conduction in a degenerate system, which occurs by band overlap, rather than thermal ionization.

Since H_2_ and probably disordered Al_2_O_3_ become poor metals with MMC, virtually all insulators with intermediate strengths probably do so as well.
